# Diflunisal versus tafamidis on neuropathy and cardiomyopathy in hereditary transthyretin amyloidosis

**DOI:** 10.1002/acn3.52158

**Published:** 2024-08-02

**Authors:** Chi‐Chao Chao, Shiou‐Ru Tzeng, Ming‐Chang Chiang, Hsueh‐Wen Hsueh, Wan‐Jen Hsieh, Yuan‐Chun Chao, Mei‐Fang Cheng, Yen‐Hung Lin, Mao‐Yuan Su, Chun‐Hsiang Huang, Yi‐Shiang Wang, Ming‐Fang Hsieh, Ping‐Huei Tseng, Sung‐Tsang Hsieh

**Affiliations:** ^1^ Department of Neurology National Taiwan University Hospital Taipei Taiwan; ^2^ Institute of Biochemistry and Molecular Biology, College of Medicine National Taiwan University Taipei Taiwan; ^3^ Department of Biomedical Engineering National Yang Ming Chiao Tung University Taipei Taiwan; ^4^ Department of Medicine National Yang Ming Chiao Tung University Taipei Taiwan; ^5^ Department of Nuclear Medicine National Taiwan University Hospital Taipei Taiwan; ^6^ Department of Internal Medicine National Taiwan University Hospital Taipei Taiwan; ^7^ Department of Medical Imaging National Taiwan University Hospital Taipei Taiwan; ^8^ Protein Diffraction Group, Experimental Facility Division National Synchrotron Radiation Research Center Hsinchu Taiwan; ^9^ Department of Anatomy and Cell Biology, College of Medicine National Taiwan University Taipei Taiwan

## Abstract

**Objectives:**

Hereditary transthyretin (TTR) amyloidosis (ATTRv) is frequently complicated by polyneuropathy (ATTRv‐PN) and cardiomyopathy (ATTRv‐CM). The long‐term efficacy of diflunisal on both polyneuropathy and cardiomyopathy in ATTRv patients, especially those with non‐V30M genotypes, has not been fully investigated and compared with that of tafamidis.

**Methods:**

We compared the structural and biochemical characteristics of A97S‐TTR complexed with tafamidis with those of diflunisal, and prospectively followed up and compared the progression of polyneuropathy and cardiomyopathy between ATTRv‐PN patients taking diflunisal and those taking tafamidis.

**Results:**

Both diflunisal and tafamidis effectively bind to the two thyroxine‐binding sites at the A97S‐TTR dimer–dimer interface and equally and almost sufficiently reduce amyloid fibril formation. Thirty‐five ATTRv‐PN patients receiving diflunisal and 22 patients receiving tafamidis were enrolled. Compared with no treatment, diflunisal treatment significantly delayed the transition of FAP Stage 1 to 2 and Stage 2 to 3 and decreased the deterioration in parameters of the ulnar nerve conduction study (NCS). The progression of FAP stage or NCS parameters did not differ between patients treated with diflunisal and those treated with tafamidis. Both diflunisal and tafamidis treatments significantly decreased radiotracer uptake on ^99m^Tc‐PYP SPECT and stabilized cardiac wall thickness and blood pro‐B‐type natriuretic peptide levels. No significant adverse events occurred during diflunisal or tafamidis treatment.

**Interpretations:**

The binding patterns of both tafamidis and diflunisal to A97S‐TTR closely resembled those observed in the wild type. Diflunisal can effectively delay the progression of polyneuropathy and cardiomyopathy with similar efficacy to tafamidis and may become a cost‐effective alternative treatment for late‐onset ATTRv‐PN.

## Introduction

Hereditary transthyretin (TTR) amyloidosis (ATTRv), caused by mutations in the TTR gene, is a systemic disease characterized by extracellular amyloid deposition caused by misfolding of the TTR protein throughout the body.^1^ The clinical phenotypes of ATTRv depend on the site of amyloid deposition, which frequently occurs in the peripheral nervous system, followed by the heart.^2^ ATTRv‐polyneuropathy (ATTRv‐PN) is an adult‐onset polyneuropathy characterized by motor weakness, impairment of all sensory modalities, neuropathic pain and dysautonomia.^1^, ^3^ Cardiomyopathy (ATTRv‐CM) is also a common phenotype associated with polyneuropathy^4^ and is a main prognostic determinant for shorter survival in ATTRv‐PN patients.^5^, ^6^ ATTRv‐PN patients usually follow a relentlessly progressive course.^7^ The prognosis of treatment‐naive ATTRv‐PN patients is poor: most patients lose ambulation ability within 5 years, and cardiac complications are the most common cause of mortality.^8^, ^9^ These observations suggest that early recognition and simultaneous management of both polyneuropathy and cardiomyopathy are critical for the prognosis of ATTRv‐PN patients.

Traditionally, treatment options for ATTRv‐PN are limited except for liver transplantation to eliminate variant transthyretin.^10^ Rapid progress has been made in the treatment of this disease in recent years, and various novel disease‐modifying therapies, including TTR stabilizers and TTR gene‐silencing agents, have shown various levels of clinical benefit by improving multisystem manifestations in patients with ATTRv‐PN.^11^, ^12^, ^13^, ^14^ These developments introduce a new era in preventing further amyloidosis and facilitating the long‐term stabilization of the disease. However, the effects of these therapies have been based on randomized placebo‐control clinical trials; whether the therapeutic efficacy of different drugs in the same category acting as a TTR stabilizer, such as diflunisal versus tafamidis, was comparable was not clear.

Diflunisal is a generic nonsteroidal anti‐inflammatory drug (NSAID), and tafamidis is a non‐NSAID benzoxazole derivative.^15^ Both drugs can successfully complex to the thyroxine binding site and kinetically stabilize TTR tetramers, inhibiting the release of the TTR monomer required for amyloidogenesis.^16^, ^17^ Previous clinical trials among ATTRv‐PN patients have shown that diflunisal and tafamidis can reduce the progression of neurological impairment.^13^, ^14^ Additionally, tafamidis can reduce the number of cardiovascular‐related hospitalizations and the rate of all‐cause mortality in patients with cardiomyopathy related to TTR amyloidosis.^18^ However, the effects of diflunisal and tafamidis on ATTRv have never been compared, and the majority of enrolled patients in previous trials had the V30M or V122I genotype. Patients with the A97S mutation, the major cause of ATTRv in Taiwan and East Asia,^8^, ^19^, ^20^, ^21^ usually manifest with late‐onset polyneuropathy and cardiomyopathy^4^ and have rarely been included in these studies.

We previously documented the binding of diflunisal to A97S‐TTR,^22^ but the structural biology of the A97S‐TTR/tafamidis complex has not been determined.^17^ In the present study, we determined the structure of A97S‐TTR in complex with tafamidis at 1.69 Å resolution as the background and rationale for the clinical study. We prospectively followed Taiwanese A97S‐predominant ATTRv‐PN patients who regularly received either diflunisal or tafamidis treatment to investigate whether these treatments could delay the progression of polyneuropathy and cardiomyopathy and to compare the efficacy of diflunisal to that of tafamidis.

## Methods

### Expression and purification of recombinant human TTR variants

Recombinant human A97S‐TTR was obtained as described previously.^22^ Briefly, we first purified TTR‐A97S protein with Ni Sepharose high‐performance affinity resin, and then we further purified these proteins using a HiLoad 16/600 Superdex 200 column. We confirmed the tetrameric forms of the TTR variants via 15% SDS–PAGE.

### Nuclear magnetic resonance (NMR) spectroscopy

The ratios of the NMR signal intensities were obtained as described previously.^22^ Briefly, we labelled isotopes by growing cells in M9 minimal medium with ^15^NH_4_Cl as the only nitrogen source. NMR samples contained uniformly 15N‐labeled A97S and either tafamidis or diflunisal, with 5% D_2_O and 5% DMSO‐d6. We acquired two‐dimensional HSQC spectra using a Bruker 800‐MHz spectrometer at 320 K and processed them with the NMRPipe program. The sequence‐specific backbone resonance assignment of TTR‐A97S was obtained from BioMagResBank (ID: 27576). We calculated and plotted the intensity ratios of the HSQC signals using the spectrum of A97S‐TTR in the presence of DMSO‐d6 as a reference, excluding overlapping peaks from the analysis. The NMR signal intensity ratio, denoted as I/I_0_ compares the intensity of the drug‐bound signals (I) to that of A97S‐TTR in the presence of DMSO‐d6 (I_0_).

### Crystallography and structure determination

The well‐shaped crystals of the A97S‐TTR/tafamidis complex were obtained as described previously.^17^ Briefly, we mixed purified TTR‐A97S protein with three times the amount of tafamidis, 18% (2‐hydroxypropyl)‐β‐cyclodextrin and 10% DMSO at room temperature. The TTR‐A97S/tafamidis cocrystal was then grown using the vapour‐diffusion hanging drop method at room temperature. The reservoir solution contained 0.05 M calcium chloride dihydrate, 0.1 M Bis‐Tris and 25% PEG monomethyl 550 ether. X‐ray diffraction data were collected at a wavelength of 0.97625 Å^2^ using beamline TPS 07A at the National Synchrotron Radiation Research Center (NSRRC) in Taiwan.

### Congo red binding assay

We employed amyloid‐specific Congo Red (CR) staining following a method described in our previous study to measure the level of amyloid.^22^ Briefly, we incubated A97S‐TTR in acidic buffer at 37°C for 6 days. After vortexing, we mixed CR dye with a 50 μL suspension of TTR variants. Using a DU730 spectrophotometer, we obtained spectra from 400 to 600 nm. Fibril quantification was calculated as μmoles of CR‐bound fibrils per litre of amyloid suspension using the following equation: [(A540/25295) − (A477/46306)] × 1000,000.

### Subjects and design

We performed a single‐centre, prospective study to investigate the outcome of patients with ATTRv‐PN who received diflunisal or tafamidis treatment and regular follow‐up at outpatient clinics at National Taiwan University Hospital. The diagnostic criteria for ATTRv‐PN were as follows: (1) transthyretin pathogenic mutation, (2) clinical evidence of sensorimotor or autonomic neuropathic symptoms and evidence of axonal polyneuropathy on nerve conduction studies or skin biopsy and (3) no monoclonal paraprotein in the serum by immunoelectrophoresis. Patients with poorly controlled diabetes mellitus, renal insufficiency, autoimmune disorders, infections, toxin exposure, vitamin B12 deficiency or malignancy were excluded. All participants received clinical evaluations, including the assessment of clinical symptoms and neurological examinations. Disability due to polyneuropathy was evaluated according to the familial amyloid polyneuropathy (FAP) stage.^23^ The laboratory studies included nerve conduction studies (NCSs) for the evaluation of polyneuropathy and echocardiography, 99mTc‐PYP SPECT imaging and blood pro‐B‐type natriuretic peptide for the evaluation of cardiomyopathy.

Patients who had ever received gene silencing therapy before using diflunisal or tafamidis were excluded. To evaluate the effects of diflunisal or tafamidis treatment, we retrieved the data of historic ATTRv‐PN patients before disease‐modifying therapies were available for comparison with the natural course of disease progression and changes in neurophysiology. This study was approved by the Ethics Committee of National Taiwan University Hospital. Written informed consent was obtained from all participants prior to all procedures being performed.

### Nerve conduction study

A nerve conduction study (NCS, Nicolet Viking, Madison, WI) was performed following established methods in a temperature‐controlled room.^24^, ^25^ The median and ulnar nerves of the upper limbs and the peroneal, tibial and sural nerves of the lower limbs were studied. A sensory nerve conduction study was performed antidromically. The amplitude of compound muscle action potential (CMAP) and sensory nerve action potential was measured from the baseline at the onset of the waveform to the negative peak. To compare the changes in NCS over time, we calculated the rate of reduction in the ulnar CMAP amplitude as (baseline CMAP amplitude − follow‐up CMAP amplitude)/(baseline CMAP amplitude × time interval between baseline and follow‐up) × 100%. The rate of slowing of the ulnar motor conduction velocity was calculated by similar formulae.

### ^99m^Tc‐PYP SPECT imaging

Planar single‐photon emission computed tomography (SPECT) imaging of the chest was performed 3 hours after intravenous injection of 20 mCi of technetium pyrophosphate (^99m^Tc‐PYP). The image acquisition parameters and protocol followed the joint guidelines established by The Taiwan Society of Cardiology (TSOC), the Society of Nuclear Medicine of the Republic of China^26^ and the multisocietal expert consensus recommendation.^27^, ^28^ The images were analysed visually and semiquantitatively based on visual scores (VS, grade 0–3)^26^ and the volumetric heart‐to‐contralateral lung ratio (H/CL ratio).^29^

### Echocardiography

An echocardiographic ultrasound system (IE33, Philips; Andover, MA) was used for echocardiography. Echocardiographic studies, including two‐dimensional, M‐mode and Doppler ultrasound recordings, were performed. Interventricular septum thickness (IVSd) and left ventricular posterior wall thickness in diastole (LVPWd) were measured according to the procedures set forth by the American Society of Echocardiography.^30^

### Statistical analysis

Numerical variables are expressed as the mean ± SD and were compared with *t*‐tests if the data followed a Gaussian distribution; otherwise, nonparametric tests were used to compare data. Fisher's exact test was used to compare categorical data. Correlations between variables were analysed by simple linear regression and multiple linear regression models with the covariance of the R^2^ model and standardized correlation coefficients. The difference in the frequency of FAP stage transitions (1 to 2 and 2 to 3) between the diflunisal treatment group and treatment‐naïve or tafamidis treatment group was evaluated with the log‐rank test. The probability of the transition from FAP Stage 1 to 2 and from FAP Stage 2 to 3 was estimated by the Kaplan–Meier failure function (complement of the Kaplan–Meier survival function). All analyses were performed using Stata software (StataCorp LP, College Station, TX) and GraphPad Prism (GraphPad Software, San Diego, CA). The results were considered significant at *p* < 0.05.

## Results

### Both tafamidis and diflunisal stabilize the TTR dimer‐dimer interface

**Figure 1:**
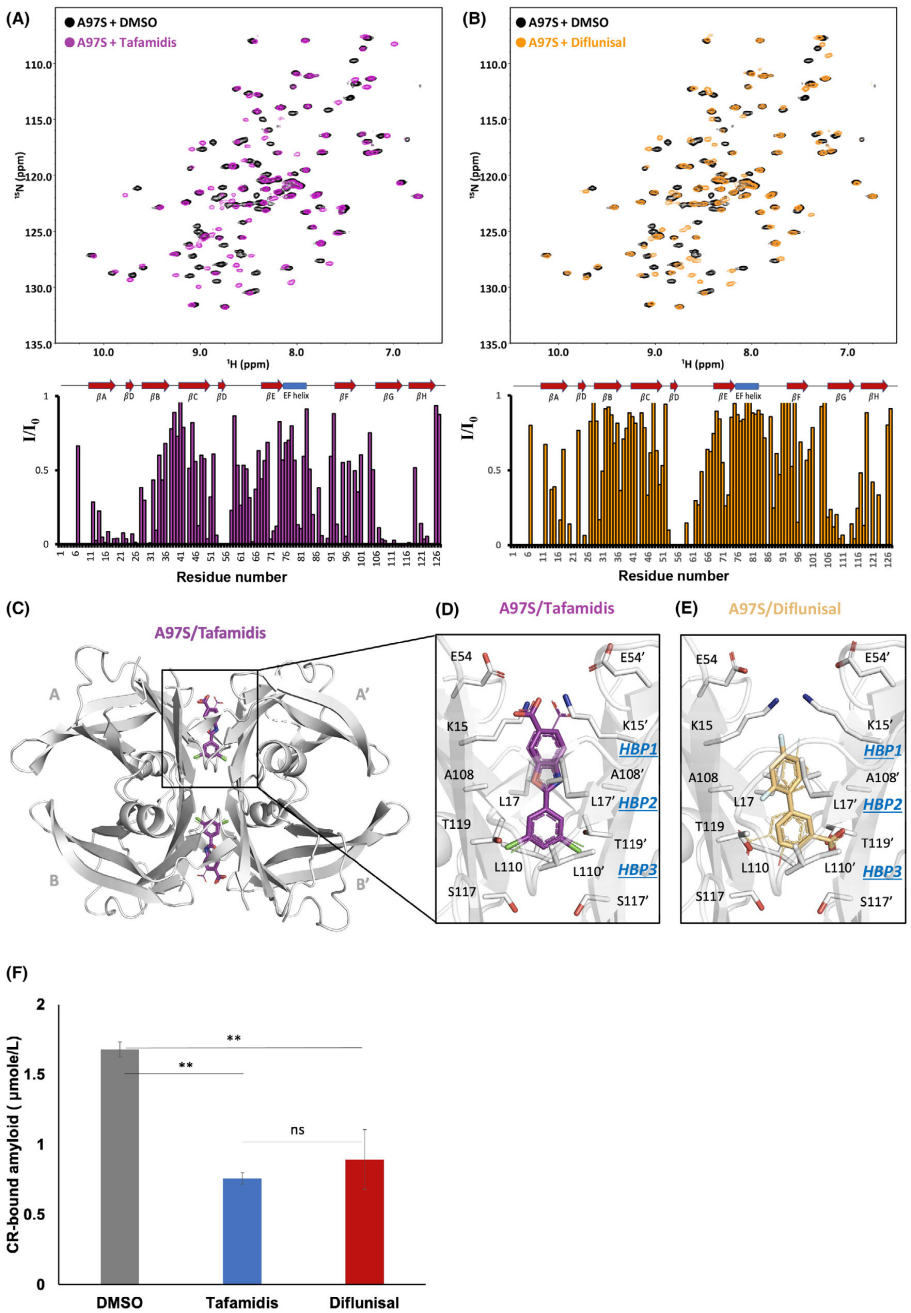
Structural analyses of stabilizer binding sites in A97S‐TTR. (A) NMR studies on tafamidis and diflunisal binding to A97S‐TTR. Comparison of 2D TROSY‐HSQC spectra: ^15^N‐labeled A97S‐TTR with (purple) and without (black) tafamidis. Intensity fluctuations observed in individual residues of A97S‐TTR upon tafamidis binding. (B) 2D TROSY HSQC spectra of 15N‐labeled A97S‐TTR with (orange) and without (black) the presence of diflunisal. Changes in the intensity of each residue of A97S‐TTR due to diflunisal binding. The ratio of NMR signal intensities, denoted as I/I_0_, refers to the signal intensity of stabilizer‐bound A97S‐TTR. (C) Crystal structures of the A97S‐TTR ligand complexes. Global view of TTR‐A97S bound to tafamidis (PDB ID: 8YQD). The overall structure is represented as a white cartoon, and tafamidis is shown as purple sticks. (D) Close‐up view of one of the tafamidis binding sites in the TTR‐A97S/tafamidis complex structure. Two different binding modes of tafamidis are shown as purple sticks and lines. (E) Two different binding modes of diflunisal are shown as orange sticks and lines. The side chains of TTR‐interacting residues are labelled and represented by white sticks. (F) Both tafamidis and diflunisal effectively inhibited the acid‐mediated aggregation of A97S‐TTR. The A97S‐TTR samples were incubated in the absence or presence of tafamidis or diflunisal at a molar ratio of 1:2 (A97S:drug) at pH 4.0 for 6 days, and the acid‐induced fibrils of A97S‐TTR were quantified using Congo red (CR) dye.

To investigate the interaction between A97S‐TTR and stabilizers, we used solution NMR to compare the binding of A97S‐TTR with tafamidis and diflunisal because these agents are known to stabilize WT‐TTR. The interactions of A97S‐TTR with tafamidis and diflunisal were examined by measuring the reduction in the NMR peak intensity using two‐dimensional [^15^N, ^1^H]‐NMR spectroscopy (Fig. 1A,B). In both cases, the resonance peaks that significantly shifted as a result of drug binding were mainly located at the dimer–dimer interface, which was previously reported as the thyroxine‐binding site. Although the complex structures of tafamidis and TTR variants (V30M, D38A, S52P, L55P and V122I) were previously determined,^17^ previous studies did not include the A97S‐TTR/tafamidis complex. Here, we characterized the cocrystal structure of tafamidis and A97S‐TTR at a resolution of 1.69 Å (Fig. 1C and Table 1). This high‐resolution crystal structure provided a clear and unambiguous depiction of the position of tafamidis within the electron density map of the TTR dimer–dimer interface. Notably, the presence of a twofold symmetry axis along the T4‐binding pocket results in two distinct binding modes of tafamidis, each related by a 180° rotation (Fig. 1D). Compared to those in the published A97S‐TTR/diflunisal complex (Fig. 1E), the chlorine atoms in the 3,5‐dichloro ring of tafamidis facilitate intersubunit bridging primarily through hydrophobic interactions between HBP3 and 3′, while its carboxylate group is indirectly connected to the ε‐amino group of K15 or K15′ through water molecules. Our structural investigations have shown that both diflunisal and tafamidis effectively bind to the two T4 binding sites located at the TTR dimer‐dimer interface, resulting in the stabilization of tetrameric A97S‐TTR. Furthermore, previous studies have shown that A97S and V30M fibrillization were much stronger than that of the WT, while the amyloid levels in A97S were similar to those in V30M when tested with a Congo Red (CR) assay at low pH. We also evaluated whether tafamidis and diflunisal inhibited the amyloidogenicity of A97S in vitro, revealing that both compounds equally and almost sufficiently reduced fibril formation (Fig. 1F). In summary, these structural and biochemical assessments indicated that both diflunisal and tafamidis effectively stabilize A97S‐TTR and provided a foundation for treating ATTRv patients with either diflunisal or tafamidis.

**Table 1:** Data collection and refinement statistics of TTR‐A97S/tafamidis.

	TTR‐A97S/tafamidis
Data collection	
PDB code	8YQD
Wavelength (Å)	0.97625 Å
Resolution range (Å)	25.54–1.692 (1.752–1.692)^a^
Space group	*P2*_*1*_ *2*_*1*_ *2*
Unit cell Parameters	
a, b, c (Å)	85.303, 42.606, 63.774
α, β, γ (°)	90, 90, 90
Total reflections	272804 (27300)^a^
Unique reflections	26616 (2596)^a^
Completeness (%)	99.70 (98.82)^a^
Average I/σ	21.10 (2.30)^a^
Multiplicity	10.30 (10.50)^a^
Refinement	
Reflections for working set	26594 (2593)^a^
Reflections for test set	1330 (130)^a^
R_work_ ^b^	0.1960 (0.3443)^a^
R_free_ ^*c*^	0.2244 (0.3492)^a^
Number of non‐hydrogen atoms	1906
Macromolecules	1754
Ligand	40
Solvent	112
RMS deviations	
Bond lengths (Å)	0.009
Bond angle (°)	1.08
Average B‐factor	36.26
Macromolecules	35.85
Ligands	30.01
Solvent	44.86

^*a*^ Statistics in parentheses are shown for the highest‐resolution shell.

^*b*^ R_work_ = ∑_hkl_ |F_o_−F_c_|/∑_hkl_ F_o_, where F_o_ and F_c_ denotes the observed and calculated structure factors of reflection hkl.

^*c*^ R_free_ is the R factor for the randomly selected 5% subset from the reflection data.

### Clinical profiles of patients with ATTRv‐PN

Table 2 shows the demographic data of the ATTRv‐PN patients included in the analysis. Thirty‐five patients (24 men), aged 65.0 ± 6.0 years, received diflunisal. All patients had TTR mutations (A97S, 33 patients; F33L, 1 patient and E89K, 1 patient) and sensorimotor and/or autonomic polyneuropathy at enrollment, with an onset age of 62.0 ± 5.9 years. Based on neurological deficits, 24 patients were at FAP Stage 1, and 11 patients were at FAP Stage 2. The dosage of diflunisal (250 mg per tablet, Gentle Pharma Co., Ltd.) was 500 mg daily in 23 patients, 375 mg daily in 4 patients and 250 mg daily in 8 patients. The duration of diflunisal use until the termination of data collection in all patients was 31.6 ± 15.3 months (range 10 ~ 73 months). Twenty‐two ATTRv‐PN patients (17 men; A97S, 21 patients and F33L, 1 patient), aged 63.1 ± 5.9 years, received tafamidis. The age at the onset of polyneuropathy was 59.5 ± 5.0 years, and based on neurological deficits at enrolment, the FAP stage was 1 in six patients, 2 in eight patients and 3 in eight patients. The dosage of tafamidis (Vyndamax, 61 mg per capsule, Pfizer, Inc.) was 61 mg once daily in all patients. The duration of diflunisal use until the termination of data collection in all patients was 35.3 ± 11.5 months (range 3–48 months).

**Table 2:** Demographic and clinical characteristics of hereditary transthyretin amyloidosis‐polyneuropathy (ATTRv‐PN) patients with diflunisal and tafamidis treatment.

	Diflunisal group	Tafamidis group
Number of patients	35	22
Age of onset	62.0 ± 5.9 years	59.5 ± 5.0 years
Age at enrollment	65.0 ± 6.0 years	63.1 ± 5.9 years
Sex (female:male)	11:24	5:17
Genotypes	p.Ala117Ser (A97S), *n* = 33	p.Ala117Ser (A97S), *n* = 21
	p.Phe53Leu (F33L), *n* = 1	p.Phe53Leu (F33L), *n* = 1
	p.Glu109Lys (E89K), *n* = 1	
FAP stage at enrollment	FAP Stage 1, *n* = 24	FAP Stage 1, *n* = 6
	FAP Stage 2, *n* = 11	FAP Stage 2, *n* = 8
		FAP Stage 2, *n* = 8
Drug doses	500 mg/day, *n* = 23	61 mg/day, *n* = 22
	375 mg/day, *n* = 4	
	250 mg/day, *n* = 8	
Duration of drug use	31.6 ± 15.3 months	35.3 ± 11.5 months

FAP stage, familial amyloidotic polyneuropathy stage; *n*, number of patients.

### The effects of diflunisal on ATTRv‐PN

**Figure 2:**
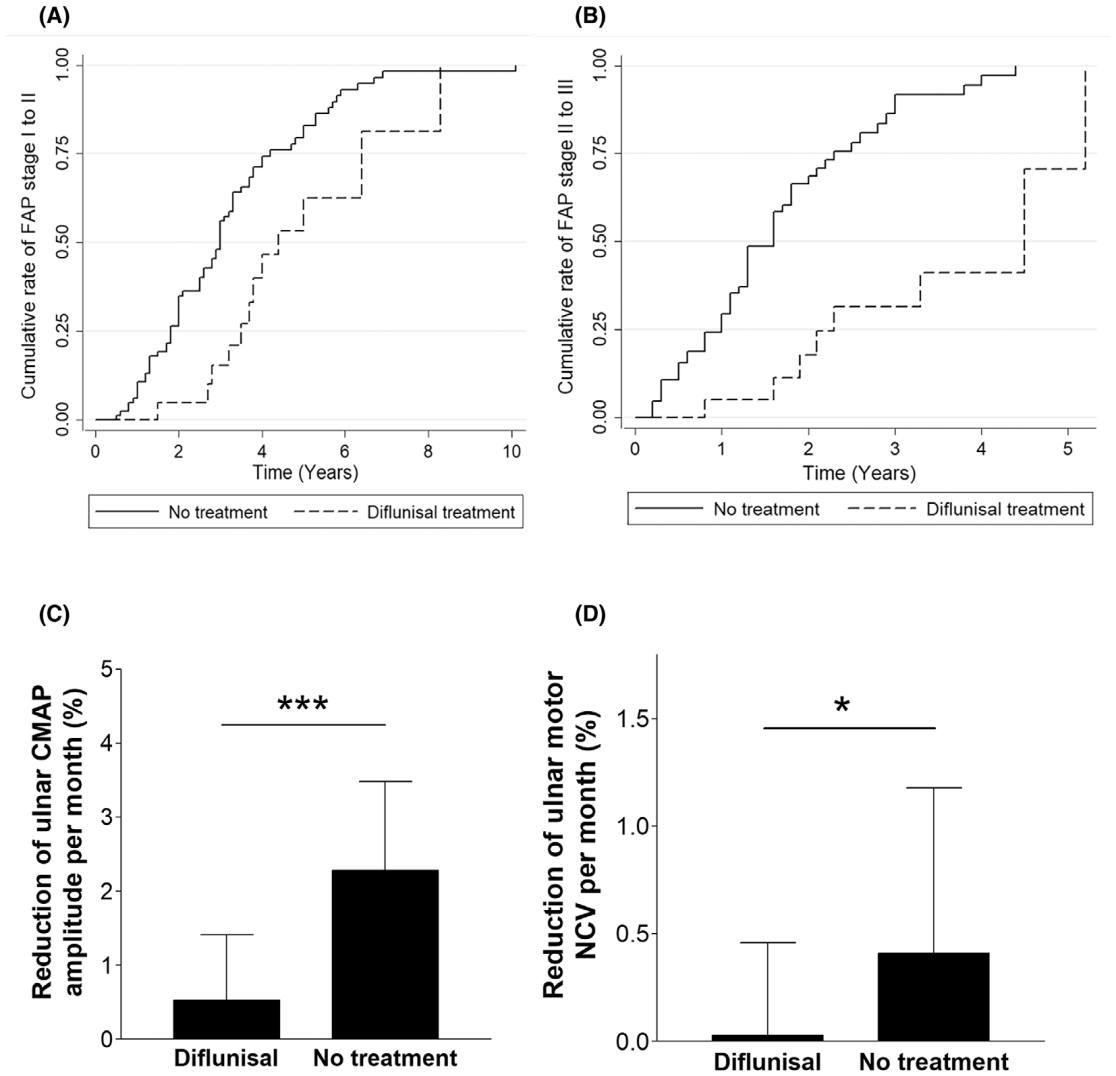
The transition from the FAP stage and the changes in ulnar motor nerve neurophysiology between ATTRv‐PN patients treated with diflunisal and those not treated. (A) The Kaplan–Meier failure curve for the cumulative portion and time interval of transition from FAP Stage 1 to 2 showed that the use of diflunisal significantly delayed the transition from FAP Stage 1 to 2 (hazard ratio [HR], 0.43; 95% confidence interval [CI], 0.23–0.79; *p* = 0.007). (B) Similarly, the use of diflunisal significantly delayed the transition from FAP Stage 2 to 3 in ATTRv‐PN patients (hazard ratio [HR], 0.18; 95% confidence interval [CI], 0.08–0.43; *p* < 0.001). (C) The reduction in the amplitude of the ulnar compound muscle action potential (CMAP) per month (%) from baseline was significantly lower in patients treated with diflunisal than in treatment‐naive patients (*p* < 0.001). (D) The decrease in the ulnar motor conduction velocity (%) per month from baseline was significantly lower in patients treated with diflunisal than in treatment‐naive patients (*p* = 0.027).

To investigate whether diflunisal can slow the progression of polyneuropathy in patients with ATTRv‐PN, we compared the transition times of FAP Stage 1 to 2 and FAP Stage 2 to 3 between ATTRv‐PN patients receiving diflunisal and historical patients without any treatment. In total, 21 patients (16 males; onset age, 61.8 ± 5.4 years) were regularly taking diflunisal during FAP Stage 1. Furthermore, 85 onset‐age‐ and gender‐matched ATTRv‐PN patients (65 males; onset age, 61.1 ± 5.9 years) without any disease‐modifying treatment were included. Figure 2A illustrates the time from the onset of FAP Stage 1 to 2 between ATTRv‐PN patients treated with diflunisal and those without treatment. Progression from FAP Stages 1 to 2 significantly differed between groups (*p* = 0.004), and diflunisal treatment significantly delayed the transition of FAP Stage 1 to 2 (hazard ratio [HR], 0.43; 95% confidence interval [CI], 0.23–0.79; *p* = 0.007). We compared 21 patients (12 males; onset age of FAP Stage 2, 66.9 ± 4.5 years) who regularly underwent diflunisal during FAP Stage 2 and 67 onset age‐ and sex‐matched patients (51 males; onset age of FAP Stage 2, 64.1 ± 6.0 years) who did not receive any disease‐modifying treatment. Figure 2B shows the time from the onset of FAP Stage 2 to 3 in the diflunisal‐treated and treatment‐naive patients. Progression from FAP Stage 2 to 3 differed between groups (*p* < 0.001), and the use of diflunisal significantly delayed the progression of FAP Stage 2 to 3 (HR, 0.18; 95% CI, 0.08–0.43; *p* < 0.001).

We further investigated whether the use of diflunisal can slow the progression of NCS parameters. The ulnar motor NCS was selected for comparison due to the low amplitude or absence of CMAPs in the nerves of the lower limbs and the confounding effects of carpal tunnel syndrome on the median nerves. Twenty‐five patients (16 males) receiving diflunisal treatment and 29 patients (23 males) not receiving any treatment were compared. Sex (*p* = 0.218), age at baseline NCS (*p* = 0.339), baseline CMAP amplitude (6.3 ± 2.4 vs. 5.1 ± 1.9 mV, *p* = 0.061) or motor nerve conduction velocity (52.2 ± 4.4 vs. 50.0 ± 4.7 m/s, *p* = 0.086) did not differ between these two groups. The use of diflunisal significantly decreased the rate of reduction in CMAP amplitude (0.53 ± 0.89 vs. 2.28 ± 1.20%/month, *p* < 0.001; Fig. 2C) and the slowing of motor nerve conduction velocity in the ulnar nerves (0.03 ± 0.43 vs. 0.41 ± 0.77%/month, *p* = 0.027; Fig. 2D).

### Comparison of diflunisal and tafamidis in ATTRv‐PN patients

**Figure 3:**
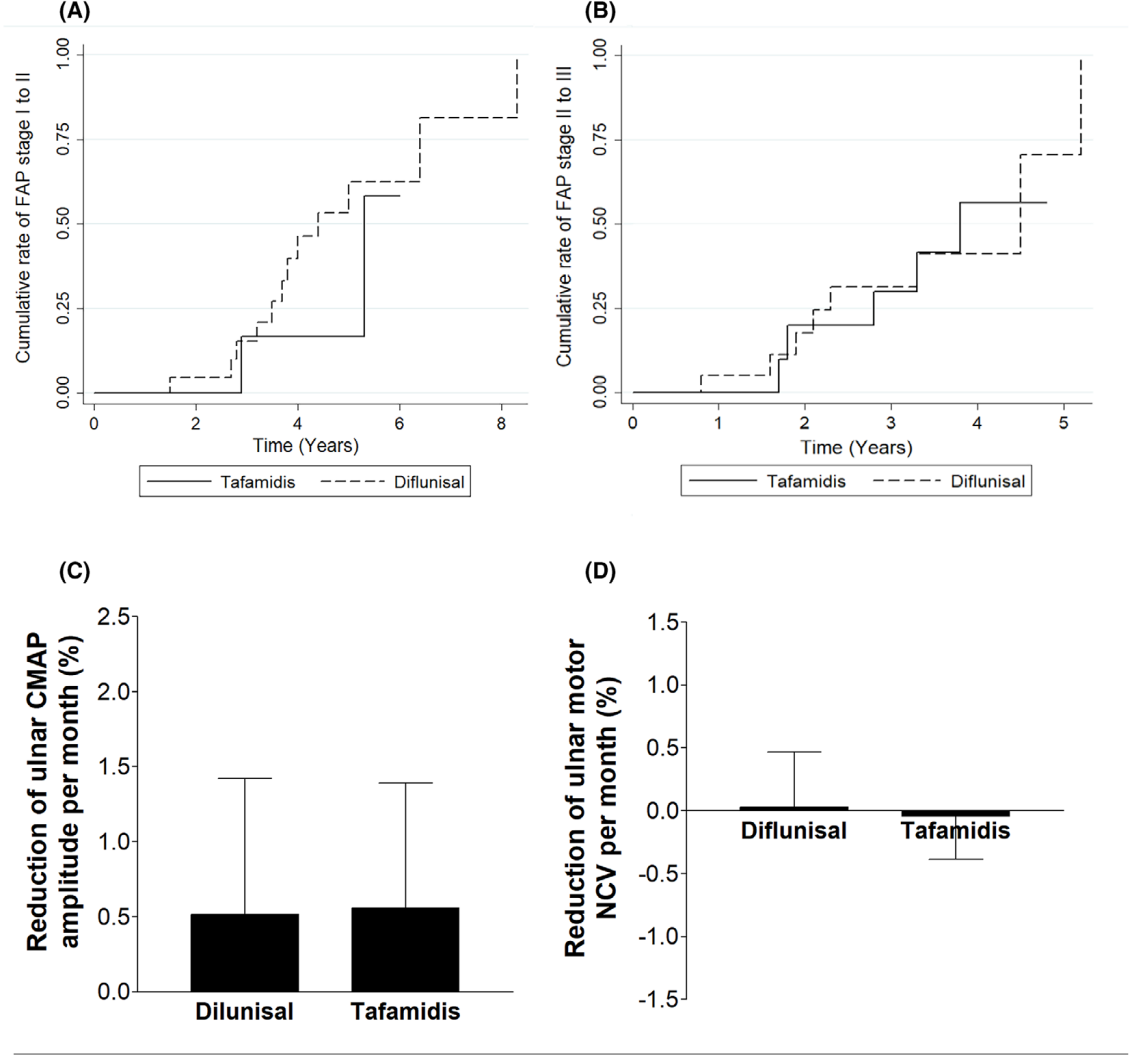
The transition from the FAP stage and the changes in ulnar motor nerve neurophysiology between ATTRv‐PN patients treated with diflunisal and those treated with tafamidis. (A) The Kaplan–Meier failure curve for the cumulative portion and time interval of transition from FAP Stage 1 to 2 showed no difference in progression between patients treated with diflunisal and those treated with tafamidis (*p* = 0.332). (B) Similarly, the progression from FAP Stage 2 to 3 did not differ between patients receiving diflunisal and those receiving tafamidis (*p* = 0.993). (C) The reduction in the amplitude of the ulnar compound muscle action potential (CMAP) per month (%) from baseline (*p* = 0.889) and (D) the decrease in the ulnar motor conduction velocity per month from baseline (*p* = 0.623) were similar in patients treated with diflunisal and tafamidis.

To compare the effects of diflunisal and tafamidis on ATTRv‐PN, we analysed the transition time of FAP Stage 1 to 2 and Stage 2 to 3 between patients receiving diflunisal and those receiving tafamidis therapy. In total, 21 patients (16 males) and 6 patients (6 males) were taking diflunisal and tafamidis, respectively, during FAP Stage 1. The onset age (*p* = 0.180) and sex (*p* = 0.180) were similar between these two groups. Figure 3A shows the progression from the onset of FAP Stage 1 to 2 between the ATTRv‐PN patients treated with diflunisal and those treated with tafamidis, which did not differ between groups (*p* = 0.332). We further compared 21 patients (12 males) taking diflunisal and 10 patients (8 males) taking tafamidis during FAP Stage 2. Age at drug administration (*p* = 0.069) or sex (*p* = 0.221) did not differ between these two groups. Figure 3B shows the progression from the onset of FAP Stage 2 to 3 between the ATTRv‐PN patients treated with diflunisal and those treated with tafamidis, which did not differ between groups (*p* = 0.993).

We also compared the effects of diflunisal and tafamidis on the progression of NCS parameters of ulnar motor nerves in 24 patients (15 males) receiving diflunisal and 14 patients (11 males) receiving tafamidis. Sex (*p* = 0.317), age at baseline NCS (*p* = 0.136), duration of treatment (*p* = 0.079), baseline CMAP amplitude (6.1 ± 2.3 vs. 4.6 ± 2.2 mV, *p* = 0.062) or motor nerve conduction velocity (51.9 ± 4.3 vs. 49.3 ± 4.8 m/s, *p* = 0.090) did not differ between these two groups. Moreover, the rate of reduction in CMAP amplitude (0.52 ± 0.90 vs. 0.56 ± 0.83%/month, *p* = 0.889, Fig. 3C) and the slowing of motor nerve conduction velocity (0.02 ± 0.44 vs. −0.04 ± 0.34%/month, *p* = 0.623, Fig. 3D) did not differ between patients treated with diflunisal and those treated with tafamidis.

### The effects of diflunisal and tafamidis on cardiomyopathy

To explore whether the use of diflunisal can stabilize or improve ATTRv‐CM, we examined changes in several parameters, including (1) radiotracer uptake in the heart on 99mTc‐PYP SPECT imaging, (2) the IVSd and LVPWd on echocardiography and (3) pro‐B‐type natriuretic peptide (pro‐BNP) before and after diflunisal treatment, and we compared these parameters to those of patients taking tafamidis (Table 3).

**Table 3:** Changes of cardiac parameters in hereditary transthyretin amyloidosis‐polyneuropathy (ATTRv‐PN) patients with diflunisal and tafamidis treatment.

	Diflunisal group	Tafamidis group
99mTc‐PYP SPECT imaging		
Number of patients	15	20
Interval of follow‐up	28.3 ± 12.5 months	37.9 ± 8.0 months
Volumetric H/L ratio		
Baseline	3.81 ± 0.88	3.77 ± 0.87
Follow‐up	3.28 ± 0.65	2.95 ± 0.51
Echocardiography		
Number of patients	17	21
Interval of follow‐up	32.4 ± 13.7 months	34.4 ± 7.2 months
IVSd		
Baseline	1.35 ± 0.29 cm	1.53 ± 0.34 cm
Follow‐up	1.44 ± 0.30 cm	1.53 ± 0.25 cm
LVPWd		
Baseline	1.31 ± 0.25 cm	1.42 ± 0.26 cm
Follow‐up	1.31 ± 0.27 cm	1.40 ± 0.21 cm
Pro‐B‐type natriuretic peptide in the blood		
Number of patients	17	21
Interval of follow‐up	26.9 ± 12.4 months	36.2 ± 10.9 months
Pro‐BNP		
Baseline	853.1 ± 641.5 pg/mL	1493.7 ± 2614.9 pg/mL
Follow‐up	783.4 ± 668.1 pg/mL	2018.7 ± 2887.6 pg/mL

99mTc‐PYP SPECT, technetium‐99m pyrophosphate single‐photon emission computed tomography; H/CL, heart‐to‐contralateral lung; IVSd, interventricular septum thickness in diastole; LVPWd, left ventricle posterior wall thickness in diastole; Pro‐BNP, pro‐B‐type natriuretic peptide.

**Figure 4:**
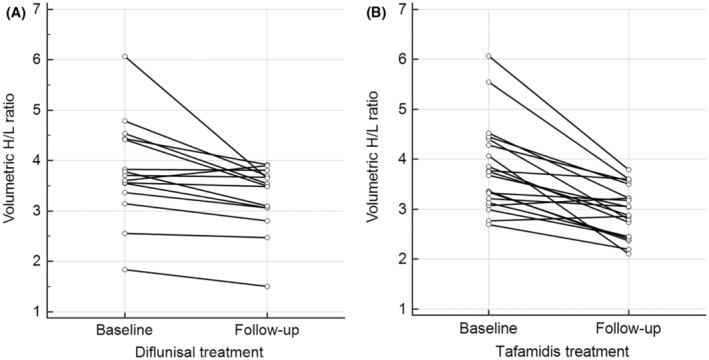
Changes in the volumetric H/CL ratio on 99mTc‐PYP SPECT imaging in ATTRv‐PN patients treated with diflunisal and tafamidis. The volumetric H/CL ratio on 99mTc‐PYP SPECT imaging decreased significantly between baseline and follow‐up after the diflunisal (A, *p* = 0.007) and tafamidis (B, *p* < 0.001) treatments.

Fifteen ATTRv‐PN patients (9 males) receiving diflunisal treatment and 20 patients (15 males) receiving tafamidis treatment were included in the 99mTc‐PYP SPECT imaging analysis. At baseline, significant radiotracer uptake in the heart was noted in all patients, with visual score grade 2 in 7 and 7 patients and grade 3 in 8 and 13 patients in these two groups of patients, respectively. The intervals between baseline and follow‐up imaging were 28.3 ± 12.5 months in diflunisal‐treated patients and 37.9 ± 8.0 months in tafamidis‐treated patients (*p* = 0.010). Diflunisal treatment significantly lowered radiotracer uptake in the heart, as indicated by a decrease in the volumetric H/CL ratio from 3.81 ± 0.88 at baseline to 3.28 ± 0.65 at follow‐up (Fig. 4A, *p* = 0.007). Similarly, tafamidis treatment significantly decreased heart radiotracer uptake from 3.77 ± 0.87 at baseline to 2.95 ± 0.51 at follow‐up (Fig. 4B, *p* < 0.001). The reduction ratio of heart radiotracer uptake (follow‐up volumetric H/CL ratio/baseline volumetric H/CL ratio) did not significantly differ between patients receiving diflunisal and those receiving tafamidis (0.88 ± 0.12 vs. 0.79 ± 0.14, *p* = 0.073).

For echocardiography analysis, 17 (10 males) and 21 (16 males) ATTRv‐PN patients receiving diflunisal and tafamidis treatment, respectively, were included. The IVSd (1.35 ± 0.29 vs. 1.44 ± 0.30 cm, *p* = 0.078) or LVPWd (1.31 ± 0.25 vs. 1.31 ± 0.27 cm, *p* = 0.965) did not differ at baseline or after diflunisal treatment, with an interval of 32.4 ± 13.7 months (range 13 to 63 months). Similarly, the IVSd (1.53 ± 0.34 vs. 1.53 ± 0.25 cm, *p* = 0.888) or LVPWd (1.42 ± 0.26 vs. 1.40 ± 0.21 cm, *p* = 0.527) did not differ at baseline or after tafamidis treatment, with an interval of 34.4 ± 7.2 months (range 15 to 42 months). The ratio of follow‐up IVSd to baseline IVSd (1.07 ± 0.16 vs. 1.01 ± 0.15, *p* = 0.255) or the ratio of follow‐up LVPWd to baseline LVPWd (1.01 ± 0.19 vs. 1.00 ± 0.14, *p* = 0.732) did not differ between patients receiving diflunisal and those receiving tafamidis.

We then compared changes in pro‐BNP between 17 diflunisal‐treated patients (10 males) and 21 tafamidis‐treated patients (16 males). The pro‐BNP at baseline or after diflunisal treatment did not differ (853.1 ± 641.5 vs. 783.4 ± 668.1 pg/mL, *p* = 0.407), with an interval of 26.9 ± 12.4 months (range 11 to 51 months). A similar pattern was noted for pro‐BNP at baseline and after tafamidis treatment (1493.7 ± 2614.9 vs. 2018.7 ± 2887.6 pg/mL, *p* = 0.445), with an interval of 36.2 ± 10.9 months (range 12 to 52 months). The ratio of follow‐up pro‐BNP to baseline pro‐BNP (1.00 ± 0.36 vs. 2.05 ± 2.44, *p* = 0.067) did not differ between patients receiving diflunisal and those receiving tafamidis.

### The safety of diflunisal

Most patients (23 patients, 65.7%) tolerated the standard dose of diflunisal (500 mg/day) throughout the observation period. The most common adverse effect was abdominal pain/gastrointestinal discomfort, which occurred in 6 patients and led to a reduction in dosage. Four patients took a reduced dose of diflunisal due to the preference of the primary care physician or patients, and 2 patients did so due to the low body weight of the patients. The follow‐up liver and renal function tests were within normal limits during the period of diflunisal use.

## Discussion

In the present study, we first demonstrated the structural biology of the A97S‐TTR/tafamidis complex and compared it with that of the A97S‐TTR/diflunisal complex. Both diflunisal and tafamidis can effectively stabilize tetrameric A97S‐TTR and inhibit its acid‐mediated aggregation. We further documented the comparable efficacy in the progression of polyneuropathy and cardiomyopathy in late‐onset ATTRv‐PN patients with predominant A97S mutation; this mutation is common in Taiwan and East Asia and shares similar phenotypes to those of the late‐onset V30M mutation.^8^ We found that (1) diflunisal treatment significantly delayed the progression of polyneuropathy, as assessed by the FAP stage and NCS, compared with treatment‐naive patients and showed similar efficacy to tafamidis treatment; (2) diflunisal and tafamidis treatments both significantly decreased heart radiotracer uptake, as evaluated by the volumetric H/CL ratio on 99mTc‐PYP SPECT, and stabilized IVSd and LVPWd on echocardiography and pro‐BNP levels in the blood. These findings suggested that diflunisal, similar to tafamidis, can effectively slow the progression of polyneuropathy and cardiomyopathy and can serve as an alternative disease‐modifying therapy for patients with late‐onset ATTRv‐PN and ATTRv‐CM.

Diflunisal, which shares the same mechanism of action with tafamidis, has been shown to have sufficient binding ability for TTR, stabilize its native tetrameric structure, and inhibit its dissociation and aggregation into amyloid.^31^ Upon daily oral administration, 20 mg tafamidis achieves a serum concentration within the range of approximately 3.4 to 6.8 μM.^32^, ^33^ In contrast, daily administration of 250–500 mg of diflunisal results in a significantly greater concentration of 100–500 μM, effectively compensating for its low affinity for the first site of the TTR protein.^22^, ^32^, ^34^ These results suggest that both stabilizers can readily interact with TTR, as the serum concentration falls within the range of 3.6–7.2 μM, reaching saturation levels.^16^ Previous research has demonstrated that diflunisal treatment can reduce the rate of disease progression, as assessed by the neuropathy impairment score (NIS), and preserve quality of life.^13^ Furthermore, diflunisal has sustained effects on both neurological and cardiac functions during long‐term administration.^35^ However, longitudinal analyses of the effects of diflunisal on ATTRv‐PN of different severities and on the progression of objective neurophysiology are lacking, especially for non‐V30M genotypes. In the present observational study, we followed up ATTRv‐PN patients who regularly received diflunisal for a mean treatment duration of 31.6 months. Compared to the natural disease course of historic ATTRv‐PN patients without any treatment, we clearly showed that the use of diflunisal significantly delayed the progression of the FAP stage regardless of whether the patients used diflunisal during FAP Stage 1 or 2. Due to the delay in the diagnosis and unavailability of diflunisal, a lag of approximately 3 years occurred from the onset of ATTRv‐PN to the treatment of diflunisal in our patients. Therefore, our study might underestimate the efficacy of diflunisal in delaying the progression of ATTRv‐PN. In addition to the clinical progression of ambulation impairment, we also followed up on the changes in ulnar motor nerve parameters in the nerve conduction study. Ulnar motor nerve parameters were chosen to avoid early ceiling effects (low amplitude or no evoked responses) in the peroneal and tibial nerves and confounding effects from prodromal carpal tunnel syndrome in the median nerve. As evidenced by NCS parameters, treatment with diflunisal significantly decreased the reduction of CMAP amplitude and the slowing of motor nerve conduction velocity in the ulnar nerves in comparison to historical treatment‐naive ATTRv‐PN patients. We also compared the above neuropathic parameters between patients treated with diflunisal and those treated with tafamidis. The progression of FAP stage transition or NCS did not differ between these two groups of patients. These findings all indicated that diflunisal treatment was associated with reduced peripheral nerve degeneration in ATTRv‐PN patients and that the efficacy of this treatment was not inferior to that of tafamidis treatment.

The heart is commonly involved in ATTRv‐PN,^36^ especially in the late‐onset phenotype.^5^, ^8^ Our previous study demonstrated that the majority of our ATTRv‐PN patients had a comorbidity of cardiomyopathy with significant radiotracer uptake in the heart on 99mTc‐PYP SPECT imaging, abnormally increased thickness of the IVSd and LVPWd on echocardiography, and increased pro‐BNP in the blood.^4^ Tafamidis has been well‐studied for treating TTR‐related cardiac amyloidosis. It has been shown to reduce cardiovascular events, all‐cause mortality and the progression of cardiac disease and is the first drug approved by the US Food and Drug Administration for ATTRv‐CM.^18^, ^37^ In the present study, both diflunisal and tafamidis treatment in patients with ATTRv‐PN and comorbid cardiomyopathy significantly decreased the volumetric H/CL ratio of the radiotracer on ^99m^Tc‐PYP SPECT during follow‐up. 99mTc‐PYP has been shown to diagnose ATTR‐related cardiomyopathy with high reproducibility and high accuracy and has been incorporated into several consensus algorithms as a noninvasive diagnostic tool for ATTR‐CM.^38^, ^39^ Because the progression of ATTRv‐CM is associated with stationary or increased radiotracer uptake on ^99m^Tc‐PYP SPECT imaging,^40^ our findings suggest the potential benefits of diflunisal and tafamidis in reducing the burden of cardiac amyloidosis. These beneficial effects were further confirmed by the findings in the present study: the thickness of the IVSD and LVPWd on echocardiography and the level of pro‐BNP in the blood were stable after diflunisal and tafamidis treatments, whereas the natural course of ATTRv‐CM was previously shown to be characterized by progressive thickening of the IVS and left ventricular wall.^41^ A comparison of the patients treated with diflunisal and tafamidis did not reveal marked differences in the changes in parameters on ^99m^Tc‐PYP SPECT, echocardiography or pro‐BNP after each treatment, suggesting the potential therapeutic role of diflunisal, like tafamidis, in ATTRv‐CM. Recent studies have shown that diflunisal treatment is associated with improved survival and the overall stability of clinical and functional cardiac biomarkers in patients with cardiomyopathy caused by wild‐type and variant TTR.^42^, ^43^ The present study further revealed that diflunisal can improve or stabilize the structural parameters of cardiomyopathy in ATTRv‐PN patients with cardiac involvement.

The present study was subject to limitations. First, the enrolled ATTRv‐PN patients were Taiwanese, and nearly all of them carried the A97S mutation. The same ethnicity and genotype in the present study may restrict the generalization of the conclusions to all ATTRv patients. Second, the progression of ATTRv‐PN was clinically evaluated based on the FAP stage, which is mainly used to assess ambulation ability and may not reflect the generalized dysfunction of ATTRV‐PN. Third, the dosage of diflunisal used in ATTRv‐PN patients was not consistent and may have confounded the comparison of therapeutic effects.

## Conclusion

Both diflunisal and tafamidis can inhibit acid‐mediated A97S‐TTR aggregation by effectively stabilizing tetrameric A97S‐TTR. Diflunisal has been proven to delay the progression of both polyneuropathy and cardiomyopathy without discernible differences in the progression of biomarkers compared to tafamidis in late‐onset ATTRv‐PN patients. Thus, diflunisal may become an obtainable, cost‐effective and practical alternative treatment for ATTRv amyloidosis in selected patients.^44^

## Author Contributions

Study conception and design Chi‐Chao Chao and Sung‐Tsang Hsieh. Acquisition of data: Chi‐Chao Chao, Hsueh‐Wen Hsueh, Wan‐Jen Hsieh, Mei‐Fang Cheng, Yen‐Hung Lin, Mao‐Yuan Su, Ping‐Huei Tseng and Sung‐Tsang Hsieh. Analysis/interpretation of data: Chi‐Chao Chao, Shiou‐Ru Tzeng, Ming‐Chang Chiang, Yuan‐Chun Chao, Mao‐Yuan Su and Sung‐Tsang Hsieh. Drafting and revision of the manuscript: Chi‐Chao Chao, Shiou‐Ru Tzeng, Ming‐Chang Chiang, Yuan‐Chun Chao and Sung‐Tsang Hsieh. Review and approval of the final version of the manuscript: all authors.

## Funding Information

This work was supported by grants from the Ministry of Science and Technology, Taiwan (107‐2314‐B‐002‐072‐MY2, 109‐2320‐B‐002‐024, 110‐2320‐B‐002‐075 and 111‐2320‐B‐002‐078 to C.‐C.C.; 110‐2320‐B‐002‐072 to S.‐T.H.) and National Taiwan University Hospital (UN109‐004 to C.‐C.C.).

## Conflict of Interest

The authors report no conflicts of interest relevant to the manuscript.

## Data Availability

The full database is available for other researchers upon reasonable request to the corresponding author.
